# Editorial: Microbial advances towards sustainable environment: Microbiome structure & integrated technologies

**DOI:** 10.3389/fmicb.2022.971696

**Published:** 2022-07-15

**Authors:** El-Sayed Salama, Byong-Hun Jeon, Junling Wang, Reda A. I. Abou-Shanab, Jiu-Qiang Xiong

**Affiliations:** ^1^Department of Occupational and Environmental Health, School of Public Health, Lanzhou University, Lanzhou, China; ^2^Department of Earth Resources and Environmental Engineering, Hanyang University, Seoul, South Korea; ^3^Department of Toxicology, School of Public Health, Lanzhou University, Lanzhou, China; ^4^College of Biological Sciences, BioTechnology Institute, University of Minnesota, St. Paul, MN, United States; ^5^College of Marine Life Sciences, Ocean University of China, Qingdao, China

**Keywords:** microbial diversity, sustainable environment, zero-waste approaches, bioremediation and biodegradation, multi-omics

Microbial diversity at the contamination sites is the major driving force behind the bioremediation of organic and inorganic matter (Cui et al., 2021; Mali et al., 2022). The multiphasic distribution of pollutants due to increased anthropogenic activities makes the bioremediation process more intricate. The microbes can break down the complex pollutants into simple degradable compounds through metabolic activity. However, the mixture of pollutants impairs the biodegradation ability of microorganisms (Singh and Kumar Mishra, 2021). The development of effective decontamination strategies can be achieved by the optimization of microbes and process parameters (Leng et al., 2020). The previously published research in microbes-based approaches has included water/soil bioremediation, waste management/ utilization, bioenergy generation, and (waste) water treatment. The increasing energy demands associated with industrialization and urbanization along with global policies for sustainable development have led to an increased focus on renewable energy projects (Kiliç and Özdemir, 2018). Recently, scientists have been working to understand and upgrade naturally available microbial processes for environmental sustainability.

The microbes are abundant in nature and their ability to transform various pollutants into nutrients or other valuable products enables them to fulfill the mission of a sustainable environment (Kaur and Gosal, 2021). The microbes can utilize contaminants as a carbon source to maintain metabolic activity and produce less complex substances which are easily degradable (Azubuike et al., 2022). Environmental factors also play a crucial role in the bioremediation of pollutants and bioenergy production (Agrawal and Verma, 2021). The microbial community composition and activity can be influenced by environmental (abiotic) conditions and biotic interactions.

In this Research Topic, microbial research has led to breakthroughs in four areas: (1) Restoration of fragile soil ecosystems, (2) Combination of soil microbes as environmentally friendly chemical alternatives, (3) Soil microbes as potential sources of industrial products, (4) Bioremediation is carried out simultaneously with biofuel production.

Under the pressure of rapid development of industrialization and huge energy demand, the exploitation of coal, petroleum, and metal resources has caused serious damage to soil surface microecology (especially microbial community), making the ecosystem fragile. Due to soil microbes' excellent nitrogen-fixing function and ability to influence soil nutrient and organic matter conversion and plant growth, their diversity contributes to the restoration and maintenance of ecosystem functions. For damaged soil, the restoration of the soil microbial community is the key to driving sustainable restoration. It was found that there was a close relationship between vegetation types and the overall structure of the soil microbial community (Peay et al., 2013; Barberan et al., 2015). Therefore, clarifying the relationship between the structure and function of soil microbial communities and vegetation types is helpful for the restoration of damaged soil ecosystems. Zhao et al. studied the structure and function of soil microbial communities in damaged mining areas under different vegetation reconstruction modes using omics tools (MiSeq high-throughput sequencing technology, PICRUSt2, and FUNGuild). It is proved that the change of vegetation reconstruction mode can lead to the change of microbial community structure and function, which provides a reference for biological restoration of a fragile ecosystem.

Furthermore, considering soil microbes play an important role in the circulation of soil elements and organic matter, the removal of pollutants, the suppression of plant pests and diseases, and the promotion of plant growth, the benefits of their diversity are not only reflected in the restoration and maintenance of soil ecosystem functions. Combinations of soil microbes may also be considered as eco-friendly alternative chemicals to increase soil fertility and promote plant growth and development, adding or enhancing functions that individual microbe does not have or are not signed for. Understanding the relationship between microbial diversity and the biochemical cycle is necessary to better play the combined role of microbes. James et al. took acidic soil as an example to study the relationship between microbial taxonomic diversity and functional diversity, as well as the relationship between microbes and depth. They deeply analyzed cell signaling pathway genes and metabolic pathways from the omics level and found the main microbes that maintain the function of the acidic soil ecosystem (James et al.). Future research is expected to explore the relationship between more soil types, vegetation types, and microbes, and give full play to the role of microbes in environmentally sustainable development.

Uncontrolled industrial production and wanton human activities in addition to the destruction of the soil ecosystem, the production of chemicals have also caused great damage to the environment. Seeking eco-friendly alternatives has become a hot topic. Once again, microbes, in addition to their function of restoring and maintaining soil ecosystems, secrete substances that can be used as eco-friendly alternatives to industrial products. Sharma et al. isolated a slime fungus, *Pyxidicoccus* sp. S252, which produces alkaline protease from the soil. Alkaline protease is known to be used in pharmaceutical, cosmetic, food, and laundry industries (Ramkumar et al., 2018). However, it is expensive and time-consuming extraction and purification process limits its large-scale application in various industries. Microbes are the preferred source of industrial enzymes due to their advantages of easy acquisition, culture, and gene manipulation. The alkaline protease isolated from *Pyxidicoccus* sp. S252 proved to have application potential in many fields. The study opens new avenues for finding potential sources of eco-friendly alternatives and promotes the ecologically sustainable development of the industry.

The wide application of plastic polymer and the complex nature of plastic makes it a major contributor to environmental pollution. Bioremediation can remove plastic to some extent and facilitate environmental sustainability. The microbial enzymes produced while the bioremediation process can not only recycle the plastic but also convert it into fuel oil (Tamoor et al.). However, it is worth mentioning that there is still a gap between current research results and future needs. Analytical techniques need to be improved, such as the combination of metagenomic sequencing and functional gene analysis. Further research is needed for commercial applications, such as the development of commercial enzymes and microbiology-based products.

Advances in microbiome-based molecular technologies have paved the way to understanding the microbial communities in their natural environments, in open, engineered ecosystems, or confined environments. Recently, integrated omics approaches have been widely used to understand specific metabolic pathways and find functional genes of microbes (Prayogo et al., 2020). A variety of functional genes (such as the genes responsible for catabolism) in microbes discovered by metagenomics sequencing have promoted the degradation mechanism of exogenous compounds (Li et al., 2021). The deep investigation of multi-omics approaches could improve the understanding of the metabolic behavior of microbes toward bioremediation and biodegradation processes. Genome sequencing and genomic libraries obtained from metagenomics analyses can effectively predict the genes. The detailed genetic information helps to explore and identify key microbial species capable of degrading specific pollutants and identify their metabolic pathways (Haque et al., 2022). The combination of highly efficient microbes would enhance the degradation efficiency of solid waste management and wastewater treatment, enable the bioremediation of novel materials, and facilitate the generation of biofuels (including biodiesel, biogas/biomethane, bioethanol, and biohydrogen). Genetic engineering of microbes promotes metabolic activity which further enhances clean energy generation (Joshi and Mishra, 2022). The mechanism of genetic engineering includes targeting the key metabolic pathways of microorganisms, either by up or down-regulation, overexpression of genes, and silencing the key gene to alter the desired metabolite content without interfering with the physiological properties of microorganisms (Ghimire et al., 2022). The selection of suitable microbes under optimal culture conditions can enhance the metabolic processes by altering the pathways and accelerating the biodegradation and bioremediation process (Tanvir et al., 2021; Azubuike et al., 2022).

The advances in omics approaches and genetic engineering tools have improved the understanding of microbial metabolic processes (de Carvalho et al., 2019). However, the large-scale applications of the bioremediation process still have some challenges such as the occurrence of emerging xenobiotics and other combined pollutants. The significant impact of biotic and abiotic factors on the microbes alters the bioremediation efficiency. The selection and separation of functional microbes with known metabolic functions is the major hurdle in the large-scale implementation of bioremediation (Ali et al., 2022; Yin et al., 2022). This research work is focused on the identification of microbes that have the potential activities and/or synergistic remediation of co-contamination; exploring the behavior of microbes in anthropogenic environments (e.g., waste treatment plants, anaerobic digestion plants, and fermentation apparatus); improving the microbes' productivity through a combination of omics and genetic engineering technologies; understand the metabolic pathways to improve biomass/biomaterials/biofuel production, and achieve zero-waste treatment using microbes and promote environmental sustainability.

With this Research Topic, recent research work and critical reviews in the field of Environmental Microbiology and Bioenergy, especially on the interactions of microbial entities during bioenergy production were reported. The major motivation of the presented work included (1) Screening of diverse ecosystems (water and soil) for potential microbes to improve environmental remediation and bioenergy production, (2) The combination of different types of microbes, (3) New biomarkers for monitoring bioremediation, (4) Waste/biomass microbial complete conversion to zero waste method, (5) Optimization of fermentation process through co-culture techniques, bio-stimulation, and technological innovation, (6) Rational designs in microbial metabolic networks in mono- and co-digestion for bio methanation, and (7) Application of novel molecular techniques (e.g., genetic tools, functional genomics, and proteomics) and adjustment and upgrading of microbial communities for improved bio-products yield.

In this Research Topic, we proposed some emerging techniques for treating pollutants in an environment that takes the interest of readers to biological remediation and biodegradation. The recently developed technology-based studies are efficient in terms of environmental protection but more in-depth studies are still required for commercialization. The incorporation and understanding of the major shortcomings in existing research would enhance the efficiency of bioremediation and biodegradation processes in near future.

## Author contributions

All authors listed have made a substantial, direct, and intellectual contribution to the work and approved it for publication.

## Funding

This work was supported by the Startup Fund for the Construction of the Double First-Class project (No. 561119201), Lanzhou University, China.

## Conflict of interest

The authors declare that the research was conducted in the absence of any commercial or financial relationships that could be construed as a potential conflict of interest.

## Publisher's note

All claims expressed in this article are solely those of the authors and do not necessarily represent those of their affiliated organizations, or those of the publisher, the editors and the reviewers. Any product that may be evaluated in this article, or claim that may be made by its manufacturer, is not guaranteed or endorsed by the publisher.
